# The effect of overuse by primary healthcare institutions on medical expenses: an empirical study from the western regions of China

**DOI:** 10.3389/fpubh.2025.1588806

**Published:** 2025-05-09

**Authors:** Yao Zhang, Yingqi Liu, Zhengxue Luo, Zhongliang Zhou, Shaoqing Gong

**Affiliations:** ^1^Department of Health Career Management and Medical Education, The Ministry of Education Key Lab of Hazard Assessment and Control in Special Operational Environment, Military Preventive Medical College, The Fourth Military Medical University, Xi’an, Shaanxi, China; ^2^China Institute for Hospital Development and Reform, Xi’an Jiaotong University, Xi’an, Shaanxi, China; ^3^Hospital Centre Office, Air Force Medical Center, Beijing, China; ^4^School of Public Policy and Administration, Xi'an Jiaotong University, Xi'an, Shaanxi, China; ^5^Luohe Medical College, Luohe, Henan, China

**Keywords:** overuse, medical expenses, unannounced standardized patient, primary healthcare services, western regions of China

## Abstract

**Background:**

Overuse stands as an important factor in the unreasonable growth of healthcare expenditure in many countries, increasing the burden of both patients and national healthcare insurance. The heterogeneity of diseases results in significant differences in clinical manifestations and treatment sensitivity among different patients with the same illness, making it urgently necessary to explore and resolve the objective assessment and measurement of overuse. This study aims to investigate the current status of overuse in primary healthcare services in China, analyze and discuss the effect of overuse on medical expenses, and draw attention to the issue of overuse.

**Method:**

The study adopted unannounced standardized patients to conduct 242 effective visits to 156 primary medical institutions in western Chinese province. The number of unnecessary medical service items reported after the visits is used to determine whether overuse exists. We set up a multiple linear regression model and used statistical methods to analyze the effect of different types of overuse on various medical expenses incurred by patients.

**Results:**

(1) Among the 242 visits, overtreatment occurred 139 times, accounting for 57.44% of the total visits. Among them, over-examination accounted for 37.41%, and over-medication accounted for 62.58%. (2) Regarding medical expenditure, the average total medical cost per capita for the 242 valid visits was 32.73 yuan, with examination fees amounting to 22.96 yuan and drug costs totaling 13.60 yuan. (3) Compared to the non-overuse group, patients in the overuse group incurred a total cost of 106.57 yuan, which was 54.33 yuan higher. The examination fees for the overuse group were 69.74 yuan, representing an additional expenditure of 18.83 yuan compared to the non-overuse patients. Drug costs for the overuse group were 39.27 yuan, an increase of 35.74 yuan compared to the non-overuse patients. Thus, overuse led to an increase of 104, 27, and 91% in total medical costs, examination fees, and drug costs, respectively. (4) For every additional unnecessary medical service item, the total medical cost will increase by 18.66 yuan, the examination cost will increase by 3.65 yuan, and the drug cost will increase by 14.48 yuan, with an increase rate of 13, 17, and 20%, respectively. For every additional unnecessary medical examination, the examination cost will increase by 11.28 yuan, with an increase rate of 13%, while there is no significant impact on total medical costs and drug costs. For every additional unnecessary drug item, the total medical cost will increase by 19.71 yuan, and the drug cost will increase by 9.81 yuan, with an increase rate of 29 and 27%, respectively, while there is no significant impact on examination costs.

**Conclusion:**

The study found that overuse is highly prevalent in primary healthcare institutions in the western provinces of China, including two types: over-examination and over-medication, with the problem of excessive medication being more prominent. Overuse has led to an increase in medical expenses, which is specifically reflected in the rise of total medical costs and drug costs. The growth in the number of unnecessary medical items is the main cause of the unreasonable rise in medical expenses.

## 1 Introduction

In recent years, with the development of China’s health care system, medical expenditure has been continuously increasing (1). From 2013 to 2023, China’s total healthcare expenses grew by nearly three times (2), and the average annual growth rate of per capita healthcare expenses (17.61%) far exceeded the growth rate of per capita disposable income (11.42%), with the proportion of healthcare expenditure in disposable income rose from 12.71 to 16.38%. Generally, the proportion of a country’s residents’ health expenditure to disposable income should not exceed 15% (3). Therefore, the economic burden of healthcare has become an obstacle for ordinary Chinese residents seeking medical and health services.

Currently, the imbalance of medical resources in China is reflected in the regional disparities between urban and rural areas as well as the differences among medical institutions of different levels. With the implementation of the national ‘Hierarchical diagnosis and treatment’ policy, the preferential policies and financial support originally intended to facilitate the development of primary healthcare institutions have been misused, resulting in a coexistence of ‘overuse’ and ‘insufficient medical care’ (4). In actual medical practices, healthcare providers may engage in overuse behavior due to pressure from performance evaluations, economic incentives, blind pursuit of advanced medical technologies, or excessive reliance on medical auxiliary diagnostic equipment. Patients may lead to over-selection of treatment plans driven by fear of illness and risk aversion. Medical administrators may contribute to frequent overuse due to the inadequacy of relevant medical systems and legal regulations. It can thus be seen that overuse is caused by multiple factors, leading to medical activities or processes that exceed the actual needs of the disease (5). Such diagnosis and treatment are excessive, unnecessary, unbeneficial, and potentially harmful to the disease (6–8).

In recent years, research on the issue of overuse at home and abroad has mainly focused on three aspects: definition and measurement, cause analysis and prevention and control, as well as medical disputes and legal improvement. Regarding definition and measurement, scholars such as Hermes DS and Wang LH (9, 10) have improved methods to identify overuse behavior through clinical guideline comparisons, expert evaluations, and big data analysis. Liu Y et al. (11, 12) have discussed cause analysis mainly from the perspectives of suppliers, demanders, and institutional factors. Prevention and control strategies include promoting clinical pathways, reforming payment methods, strengthening medical ethics education, and advancing the hierarchical medical system (13, 14). In terms of legal relationships, medical disputes arising from over-medical treatment have occurred frequently in recent years. Studdert D M and Li L et al. (15, 16) pointed out that the continuous improvement of the legal system will play a key role in preventing and punishing overuse, including improving the determination of medical responsibility, strengthening the supervision of medical practices, and improving the mechanism for patients to safeguard their rights.

This study mainly investigates the relationship between overuse and medical expenses. Domestic and international research indicates that overuse is a global issue. It leads to a waste of medical resources and increased medical expenses, significantly adding to the national health insurance burden and the economic burden of patients’ illnesses. Scholar Wieteke (17) believed that the overuse of medications and unnecessary medical procedures exacerbate the burden on the healthcare system, leading to rising medical costs; Van Dijk C. E et al. (18) compared medical service data before and after the reform of the payment system for general practitioners in the Netherlands, finding that doctors might engage in overuse for certain patients from a profit-driven perspective. Chinese scholar Ren HL (19) believed that inadequate financial compensation is the main reason for overuse in China. Government financial subsidies generally account for only 7 to 8% of public hospital revenues, or even lower (20). Their outpatient and inpatient income must cover over 90% of hospital operating expenses (21). Although the government’s financial subsidies to the primary level are continuously increasing, it is difficult to offset the income losses caused by policies such as zero profit margins on drugs for medical institutions (22). Overuse has become a routine channel for primary healthcare institutions to compensate themselves (23). At the doctor level, Lei P et al. (24) believed there is an information asymmetry between doctors and patients in medical activities. Doctors use their knowledge to gain an advantage in this information gap, thereby inducing patient demand and increasing medical expenses. At the patient level, the blind consumption of medical services by patients is one of the factors contributing to the unreasonable increase in medical expenses. Some scholars have found that under the premise of moderate medical care, 40% of patients agree to additional medical services due to psychological needs for their health security; nearly two-thirds of patients actively request unnecessary examinations and treatments (25). There are also scholars who point out that (26) overuse is one of the factors leading to the increase in medical expenses from 10 billion yuan 40 years ago to 9 trillion yuan today. It is caused by a combination of factors at multiple levels, including patients, doctors, hospitals, and society. China Health Insurance Research Association (27) mentioned that wasteful care accounts for 30% of healthcare costs. Although overuse is not explicitly mentioned, wasteful care is closely related to overuse, thus, to some extent, also demonstrating the effect of overuse on medical expenses.

In summary, the research on overuse mostly focuses on its macro-level harms, lacking more specific quantitative studies on the economic losses incurred by individual patients. The current health policy in China emphasizes a ‘grassroots-oriented’ approach. Therefore, this article aims to conduct research on primary medical institutions in the western regions of China using the anonymized standard patient method. By measuring and analyzing the over-medical treatment occurring during the diagnosis and treatment process in sample institutions, understand the current situation of over-medical treatment in grassroots medical services and its adverse effects on medical expenses. We hope to provide a certain reference basis for protecting patients’ vital interests and promoting the healthy development of grassroots medical and health services in China.

## 2 Methods

### 2.1 Research subjects and sampling methods

The research subjects of this study are doctors from primary healthcare institutions in Shaanxi Province, located in the western region of China. Specifically, they include practicing physicians and assistant physicians from community health service centers (stations) and township hospitals, as well as certified rural doctors and health workers who are actively employed. Additionally, the study includes clinicians holding practice certificates in general practice, internal medicine, obstetrics and gynecology, and pediatrics from primary and secondary hospitals. A two-stage stratified sampling method was employed in the study, where 156 primary healthcare institutions were selected, and 264 doctors were interviewed.

### 2.2 Research methods

This study conducted research on primary medical units through the anonymous standardized patient method. The main sources of test cases were selected from the ‘China National Health Service Survey and Analysis Report’ (28), and the ‘Catalogue of Basic Diseases and Standards of Rescue and Treatment Capability of Primary Healthcare Institutions (Township Health Houses and Community Health Service Centres) in China’ (29) released by the National Health and Wellness Commission of China in 2018, which explicitly includes 66 basic diseases such as internal medicine, surgery, gynaecology, and so on. Combining rich clinical experience at the grassroots level and drawing on existing surveys, the expert team selected 11 diseases from the catalogue, namely postpartum depression, hypertension, diabetes, common cold, paediatric diarrhoea, angina pectoris, gastritis, asthma, urinary incontinence, low back pain, and migraine. It carried out the design and development of the case script. The test cases were selected based on the following criteria: (i) they are relatively common in the working environment of primary care; (ii) the symptoms of the diseases are distinctive enough that the USP can be interpreted through performance; (iii) the doctor can usually obtain the initial diagnosis by listening to the patient’s chief complaint during the consultation, and the probability of invasive examination is low to a certain extent.

Unannounced standardized patients (USP) are non-medical professionals who have undergone specialized training by the research team to realistically portray the clinical symptoms of specific diseases (30). The recruitment and training of USP constituted a profound aspect of the preliminary phase of this study. Following the completion of the case scenarios, the research team recruited a total of seven USP in Shaanxi Province, adhering to the criteria outlined below by the study’s requirements. The standards included: (i) good physical health; (ii) personal characteristics matching the age, gender, and physical attributes of the USP specified in the case scenarios; (iii) ability to undergo harmless examinations conducted by doctors; (iv) capacity to authentically portray patient behaviors; (v) proficiency in objectively providing feedback and completing all information on the ‘Primary Healthcare Quality Evaluation Form’. The research team allocated the case scenarios based on the age, gender, and physical attributes of the actors. The training encompassed disease explanations, case-based teaching, and case simulations, culminating in an assessment after 8 days of intensive training.

In terms of quality control, actors who could complete four consecutive simulated visits with a line accuracy rate of 90% were considered qualified for the official survey. If the line accuracy rate was unsatisfactory during the assessment, additional line training and simulated visits were required until the desired accuracy was achieved. During the official survey, if a USP’s line accuracy rate fell below the 90% standard, their participation was suspended, and they were required to undergo retraining until they met the criterion. Simultaneously, the actors exhibited consistent demographic, disease, and emotional characteristics to different healthcare professionals, controlling for disease heterogeneity and enhancing the comparability of healthcare service quality across various contexts.

During the official survey, each standardized patient was paired with a researcher to form a survey team. The standardized patients simulated the emotions and behaviors of real patients, going through the complete diagnosis and treatment process including registration, waiting, and consultation, collecting information on medical services, costs, and healthcare experiences. After the visit, both the standardized patient and the surveyor filled out the ‘Medical Service Quality Collection Form’ (see Table 1) to objectively evaluate the quality of medical services immediately. This ‘immersive simulation’ can effectively avoid the observer effect, reduce recall bias, and restore a real diagnosis and treatment process to the greatest extent possible (31). The research process underwent strict quality control, ensuring strong credibility and explanatory power of the data.

**Table 1:** Basic contents of medical service quality collection form.

*Part One: Information on Medical Institutions*		
Basic information about medical institutions, department setup, equipment and facilities, and the condition of the medical institution environment		
*Part Two: Information on Attending Doctors and Consultation Rooms*		
Information on attending doctors, hardware configuration of consultation rooms, personnel configuration of consultation rooms, and the condition of consultation room environments		
*Part Three: Medical Quality Assessment Information*		
Indicator name	Measurement method	Evaluation method
Consultation completion rate	Consultation and examination list	Total number of doctor consultation entries/Total number of consultation entries
Examination completion rate	Consultation and examination list	Total number of doctor examination entries/Total number of examination entries
Diagnostic accuracy	List of diagnostic results	Entirely correct: all diagnostic results are correct diagnoses
		Partially correct: some diagnostic results are correct diagnoses.
		Incorrect diagnosis: all diagnostic results are incorrect diagnoses
Treatment correctness	List of treatment and disposal measures	Entirely correct: all treatment and disposal measures are correct
		Partially correct: treatment and management measures are partially correct
Patient-centred	PPPC-RC Scale	1–4 points, the lower the score, the better the patient experience
Waiting time	Consultation time record sheet	USP Time from entering the medical institution to the first time seeing a doctor
Consultation time	Consultation time record sheet	Time from starting the conversation with the doctor to the first time leaving the consultation room; if there are multiple conversations, it is the total conversation time
Consultation fee	Medical expense collection form	Total of all treatment costs
Drug fee	Medical expense collection form	Total of all medication costs
Examination fee	Medical expense collection form	Total examination costs

### 2.3 Research methodology

This study employs a multiple linear regression model to analyze the relationship between overuse and medical expenses. The variables included in the model are explained as follows:

(1) Explanatory variables

To quantify the effect of different types of overuse behaviors on medical expenses, this study incorporates three binary variables as explanatory factors: over-medical treatment, over-examination, and over-medication. Specifically, over-medical treatment refers to whether a patient has received unnecessary medical services, including redundant examinations and medications. Over-examination indicates whether any unwarranted diagnostic procedures have been performed. We refer to the latest authoritative clinical medical diagnostic guidelines, which explicitly label certain unnecessary or harmful examination items for specific diseases and combine the practical experience of primary care physicians to determine the presence of excessive examination.
Overuse refers to the administration of any unnecessary medical treatment, primarily manifesting in prolonged medication use, off-label drug use, and insurance indication drug use (32), indicating non-standard practices by the medical side in drug selection, dosage, or combination therapy, which harms patient rights (33). Currently, there is no unified standard for the classification and evaluation of ‘overuse’. In previous related studies, the most detailed classification method comes from the ‘Overuse Indicator System (First Edition)’ developed by Zheng SZ (34) from the Shanghai Medical Insurance Association in China. This system divides overuse into 13 secondary indicators and 35 tertiary indicators, such as Fragmented prescriptions, Excessive prescribing, Medication without indications, Off-label drug use, etc. This classification method is a consensus formed by national health institutions, medical insurance departments, clinical, and academic experts, with a certain degree of authority, and suggests that the classification and evaluation standards of the over are open and need continuous updating. In the realm of evidence-based medicine, the evidence provided by evidence-based medicine classifies the medical side’s diagnostic measures and medication recommendations into Class I recommendations, Class II recommendations (IIa, IIb), and Class III recommendations (35). Class I recommendations indicate that, according to current evidence-based medicine evidence or general opinion, a certain diagnostic measure or medication recommendation is considered beneficial, useful, and effective. Class II recommendations indicate inconsistent evidence-based medical results for a certain diagnostic measure or medication recommendation, with differing expert opinions. Among them, Class IIa indicates that, based on most evidence-based medical evidence or expert opinions, the diagnostic measure is considered beneficial and effective, while Class IIb indicates that, based on current evidence-based medical evidence and expert opinions, the effectiveness and benefits of the diagnostic measure are insufficient. Class III recommendations indicate that, based on current evidence-based medicine evidence and expert opinions, a certain diagnostic measure or medication recommendation is considered unbeneficial, useless, or even harmful and should be avoided. Class I recommendations have important reference value and significance for the medical side in adopting certain diagnostic measures or medication behaviors, while Class III recommendations are deemed inappropriate or prohibited, serving as a warning in medical practice.
Based on the above content, this study, referencing existing research on the classification methods of overuse, combines expert consultation and evidence-based medicine to classify medications that may be prescribed for a certain disease into five categories according to authoritative guidelines. Category one medications are recommended drugs, category two medications refer to alternative drugs, category three medications refer to ancillary drugs, category four medications refer to drugs that are completely useless but harmless, and category five medications refer to harmful drugs. In this study, we consider category four and five medications, as well as the standalone prescription of category three medications, as instances of overuse.
This study involves 11 common diseases at the primary care level. Our criteria for determining whether ‘overuse’ has occurred for each disease are based on expert opinions provided by doctors and pharmacists with more than 10 years of clinical experience from the expert team of the China Primary Health Care Quality Cohort Project (ACACIA, Primary Health Care Quality Cohort In China study) on which this study relies, as well as the text recommendations of authoritative guidelines. These specifically include the latest domestic and international medication guidelines for 11 common diseases at the primary care level, the National Essential Medicines List, the National Negotiated Medicines, and the National Centralized Volume-Based Procurement Medicine List, among other national official policy texts (as of January 2025). It also incorporates expert clinical experience and the latest clinical research conclusions. See Table 2 for details.
Furthermore, this study also measures the variations in overuse through count variables, including the number of unnecessary medical services, unnecessary examination items, and unnecessary pharmaceutical items.

(2) Dependent variable

The dependent variable of this study’s multiple linear regression model is medical expenses, including total expenditures, examination, and medication costs. To further investigate the trends in various costs, we utilize logarithmic transformations of total medical costs, examination costs, and drug expenses as metrics. When there is a nonlinear relationship between medical expenses and influencing factors, logarithmic transformation can convert the nonlinear relationship into a linear one, facilitating analysis within the regression model and thereby enhancing the model’s explanatory power.
Specifically, we measure these expenses using six indicators: total medical costs, examination costs, drug expenses, the logarithmic total medical costs, the logarithmic examination costs, and the logarithmic drug expenses.

(3) Control variables

Include 13 variables across five categories: medical institutions, doctors, medical quality, patients, and diseases (see Table 3).

**Table 2:** Reference basis for evaluating overuse in various diseases.

Serial number	Disease name	Domestic and international medication guidelines	Issuing institution	Access information
1	Postpartum depression	Guidelines for the Prevention and Treatment of Depressive Disorders in China (Second Edition); 《Management of Depression in Women: ACOG Clinical Practice Guideline》	Psychiatry Branch of the Chinese Medical Association; American College of Obstetricians and Gynecologists (ACOG)	Chinese Journal of Psychiatry; ACOG Official Website
2	Hypertension	Guidelines for the Prevention and Treatment of Hypertension in China (2024); 《ISH Global Hypertension Practice Guidelines 2023》	National Center for Cardiovascular Diseases, Chinese Hypertension League; International Society of Hypertension (ISH)	National Center for Cardiovascular Diseases; ISH Official Website
3	Diabetes	Guidelines for the Prevention and Treatment of Type 2 Diabetes in China (2024); 《Standards of Medical Care in Diabetes—2025》	Diabetes Society of the Chinese Medical Association; American Diabetes Association (ADA)	Official Website of the Chinese Journal of Diabetes; ADA Official Website
4	Common cold	Expert Consensus on the Standardized Diagnosis and Treatment of the Common Cold (2022); 《Common Cold: NICE Clinical Knowledge Summaries》	Respiratory Society of the Chinese Medical Association; National Institute for Health and Care Excellence (NICE)	Clinical Guidelines of the Respiratory Society of the Chinese Medical Association CTS; NICE official website
5	Pediatric diarrhea	Guidelines for the Diagnosis and Treatment of Acute Infectious Diarrheal Diseases in Children (2020); 《WHO Guidelines on the Management of Diarrhoea(2023)》	National Health Commission; World Health Organization (WHO)	Notice from the National Health Commission on Issuing the Guidelines for the Diagnosis and Treatment of Acute Infectious Diarrheal Diseases in Children; WHO official website
6	Angina pectoris	Guidelines for the Diagnosis and Treatment of Stable Coronary Artery Disease (2024); 《2024 ESC Guidelines on Chronic Coronary Syndromes》	Chinese Medical Association Cardiovascular Branch; European Society of Cardiology (ESC)	Chinese Journal of Cardiovascular Diseases; ESC Official Website
7	Gastritis	Guidelines for the Diagnosis and Treatment of Chronic Gastritis in China (2022); 《ACG Clinical Guideline: Management of Gastritis》	Chinese Society of Gastroenterology, Chinese Medical Association; American College of Gastroenterology (ACG)	Chinese Digestive Web - Official Website of the Chinese Society of Gastroenterology, Chinese Medical Association; ACG Official Website
8	Asthma	Guidelines for the Prevention and Treatment of Bronchial Asthma (2020); 《GINA Global Strategy for Asthma Management》	Asthma Group, Chinese Society of Respiratory Diseases, Chinese Medical Association; Global Initiative for Asthma (GINA)	Guidelines for the Prevention and Treatment of Bronchial Asthma - Chinese Journal of Tuberculosis and Respiratory Diseases; GINA official website
9	Urinary incontinence	Guidelines for the Diagnosis and Treatment of Female Stress Urinary Incontinence (2017); 《IUGA/ICS Joint Report on Female Urinary Incontinence》	Chinese Medical Association, Obstetrics and Gynecology Branch; International Urogynecological Association (IUGA) and International Continence Society (ICS)	Guidelines for the Diagnosis and Treatment of Female Stress Urinary Incontinence- Chinese Journal of Obstetrics and Gynecology; IUGA official website
10	Low back pain	Guidelines for the Diagnosis and Treatment of Acute and Chronic Non-specific Low Back Pain in China (2023); 《NICE Guideline NG193: Low Back Pain and Sciatica》	Chinese Medical Association, Orthopedics Branch; National Institute for Health and Care Excellence (NICE)	Chinese Journal of Orthopedics official website; NICE official website
11	Migraine	Chinese Guidelines for the Prevention and Treatment of Migraine (2022); 《The International Classification of Headache Disorders, 4th Edition (ICHD-4)》	Neurology Branch of the Chinese Medical Association; International Headache Society (IHS)	Chinese Guidelines for the Diagnosis and Treatment of Migraine - Neurology Branch of the Chinese Medical Association; IHS Official Website

National official policy documents: National Essential Medicines List, National Negotiated Medicines, and National Centralized Procurement List of Medicines.

**Table 3:** Types and assignments of control variables.

Serial number	Variable name	Variable type/assignment
1	Profitability of Medical Institutions	0 = Non-profit; 1 = Profit
2	Level of Medical Institutions	1 = Hospitals, Community Health Service Centers (stations), Health Centers; 2 = Outpatient Departments, Clinics, Infirmaries, Village Health Rooms
3	Doctor’s Gender	0 = Male; 1 = Female
4	Doctor’s Age	1 = <30 years old; 2 = 30 ≤ x < 50 years old; 3 = ≥50 years old
5	Doctor’s title	1 = Chief physician/Associate chief physician; 2 = Attending physician; 3 = Resident physician or no title
6	Doctor’s education	1 = University and above; 2 = Junior college/Technical secondary school; 3 = High school and below
7	Correct diagnosis	0 = Correct; 1 = Incorrect
8	Necessary item inquiry rate	Continuous variable (%)
9	Available examination service items	Continuous variable (items)
10	Consultation time	Continuous variable (minutes)
11	Disease types	1 = Postpartum depression; 2 = Hypertension; 3 = Diabetes; 4 = Cold; 5 = Pediatric diarrhea; 6 = Angina pectoris; 7 = Gastritis; 8 = Asthma; 9 = Urinary incontinence; 10 = Lower back pain; 11 = Migraine
12	Patient gender	0 = Male; 1 = Female
13	Patient region	1 = Guanzhong; 2 = Northern Shaanxi; 3 = Southern Shaanxi

## 3 Results

### 3.1 Basic situation of the research

#### 3.1.1 Basic situation of the interviewed doctors

The results indicate that after excluding failed interviews and invalid data, young male doctors aged between 30 and 50 years old constituted the majority of survey respondents, accounting for more than 60% of the total. In terms of highest educational attainment, doctors with a bachelor’s degree or above accounted for approximately 35.9% of the total, while those with college or technical secondary school qualifications represented the highest proportion, at 38.84%. Regarding professional titles, chief physicians, attending physicians, and resident physicians each accounted for approximately one-third of the total. Among them, resident physicians were relatively the most numerous, representing 36.36% of the total.

#### 3.1.2 Current situation of overuse behavior

By analyzing the records of standardized patients during their medical visits, we tallied the instances of unnecessary medical services, examinations, and medications. The results indicated that out of 242 visits, there were 139 cases of overuse, representing a frequency of 57.44%. Among all instances of overuse, excessive examinations accounted for 37.41%, while overprescription of medications constituted 62.58%. In terms of the average number of overuse items per visit, it was found to be 1.68, with 0.54 items attributed to excessive examinations and 1.36 items to overprescription of medications (See Table 4).

**Table 4:** Investigation of overuse.

District	Overuse		Excessive examination		Excessive medication	
	Frequency (Rate)	Item	Frequency (Rate)	Item	Frequency (Rate)	Item
Guanzhong	63 (26.03%)	1.92 ± 1.12	23 (9.50%)	1.26 ± 0.67	40 (16.53%)	1.51 ± 1.01
Northern Shaanxi	36 (14.88%)	1.88 ± 0.78	9 (3.72%)	1.11 ± 0.31	27 (11.16%)	1.63 ± 0.78
Southern Shaanxi	40 (16.53%)	1.92 ± 0.77	20 (8.26%)	1.24 ± 0.25	20 (8.26%)	1.40 ± 0.67
Total	139 (57.44%)	1.68 ± 0.39	52 (21.49%)	0.54 ± 0.50	87 (35.95%)	1.36 ± 0.87

### 3.2 Effect of overuse on medical expenses

#### 3.2.1 Basic situation of medical expenses

In this study, the average total medical cost per capita was 32.73 yuan, with an average inspection cost of 22.96 yuan and an average medication cost of 13.60 yuan. The difference between the sum of the cost of tests and medicines, and the total cost, is the registration fee for each consultation. A comparison of the total medical costs, inspection costs, and medication costs between the overuse group and the non-overuse group revealed that the overuse group incurred higher total medical costs, inspection costs, and medication costs than the non-overuse group. Detailed information is provided in Table 5.

**Table 5:** Comparison of medical expenses.

Project classification		Non-overuse group		Overuse group		*t* value	*p*-value
		Average cost (Yuan)	Standard deviation	Average cost (Yuan)	Standard deviation		
Overuse	Total cost	8.45	1.60	60.70	6.34	−6.96	<0.01
	examination fee	3.41	1.29	21.63	5.88	−2.63	<0.01
	Medication fee	3.67	1.02	37.24	3.82	−7.42	<0.01
Excessive examination	Total cost	26.85	2.92	80.88	14.18	−5.83	<0.01
	examination fee	3.48	0.93	51.51	14.61	−6.13	<0.01
	Medication fee	22.11	2.74	25.71	5.70	−0.59	0.551
excessive Medication	Total cost	10.40	2.00	62.24	6.63	−6.96	<0.01
	Examination fee	3.32	1.21	22.82	6.23	−2.85	<0.01
	Medication fee	5.54	1.52	37.70	3.97	−7.11	<0.01

#### 3.2.2 Effect of overuse on medical expenses

The regression results indicate that different types of overuse have varying impacts on total medical expenses, examination costs, and drug expenses. There is a statistically significant difference in medical costs between the overuse group and the non-overuse group (*p* < 0.01). Compared to non-overuse patients, overmedicalized patients incur a higher total cost of 54.32 yuan, a higher examination cost of 18.83 yuan, and a higher drug cost of 35.74 yuan, representing increases of 104, 27, and 91%, respectively. Over-examination is significantly correlated with total medical expenses and examination costs at the 0.01 level. Compared to patients without over-examination, those with over-examination have a higher total medical cost of 43.25 yuan (an increase of 62%) and a higher examination cost of 37.54 yuan (an increase of 48%), while there is no significant difference in drug costs. Over-medication is significantly correlated with total medical expenses, examination costs, and drug expenses at the 0.01 level. Overmedicalized patients have a higher total medical cost of 51.84 yuan, a higher examination cost of 19.47 yuan, and a higher drug cost of 32.16 yuan compared to non-overmedicalized patients, representing increases of 105, 29, and 89%, respectively (see Table 6).

**Table 6:** Basic regression results on the relationship between different types of overuse and costs (*N* = 242).

IV	Overuse group		Over-examination group		Over-medication group	
DV	Amount of cost increase (yuan)	Percentage of cost increase (%)	Amount of cost increase (yuan)	Percentage of cost increase (%)	Amount of cost increase (yuan)	Percentage of cost increase (%)
Total cost	54.32	104	18.83	27	35.74	91
Examination fee	43.25	62	37.54	48	– (*p*>0.01)	–
Medication fee	51.84	105	19.47	29	32.16	89

IV, Independent Variable; DV, Dependent Variable.

#### 3.2.3 Effect of the number of unnecessary medical service items on medical expenses

The Intensive Margin can make the trend of changes in medical expenses more transparent by analyzing the growth of medical expenses (36). The results show that for every additional unnecessary medical service item, there is a significant increase in total medical expenses, examination costs, and drug costs. There is a significant correlation at the 0.01 level between unnecessary medical services and total medical expenses, examination costs, and drug costs. Specifically, for every additional item of excessive medical service, total medical expenses increase by 18.66 yuan (13%), examination costs rise by 3.65 yuan (17%), and drug costs grow by 14.48 yuan (20%). Excessive examination items are significantly correlated with examination costs at the 0.05 level. For every additional unnecessary examination item, examination costs increase by 11.28 yuan (13%), while there is no significant impact on total medical expenses and drug costs. The number of excessive medication items is significantly correlated with total medical expenses and drug costs at the 0.01 level. For every additional unnecessary medication, total medical expenses increase by 19.71 yuan (29%) and drug costs increase by 9.81 yuan (27%), with no significant effect on examination costs (see Table 7).

**Table 7:** Basic regression of overuse items and medical expenses.

Item	Total cost (1)	Examination fee (1)	Medication fee (1)	Total cost (2)	Examination fee (2)	Medication fee (2)	Total cost (3)	Examination fee (3)	Medication fee (3)
Overuse item	18.66***	3.65***	14.48***						
	0.13***	0.17***	0.20***						
Excessive examination item				12.04*	11.28**	1.43			
				0.24	0.13**	0.09			
Excessive medication item							19.71***	9.63	9.81***
							0.29***	0.30	0.27***

*, **, and *** indicate significance levels of 10, 5, and 1%, respectively. Values in parentheses are robust standard errors.

## 4 Discussion

### 4.1 The phenomenon of overuse is prevalent in primary healthcare institutions

The implementers of over-medical treatment are primarily young and middle-aged male grassroots doctors with college degrees. More than half of medical activities involve over-medical treatment, and the problem of over-medication is more prominent than over-examination, regardless of whether the medical institution is located in northern Shaanxi, central Shaanxi, or southern Shaanxi. Some grassroots doctors have long had the undesirable practice of relying on antibiotics and painkillers for ‘quick treatment’. Simultaneously, due to limited educational backgrounds and regional conditions, some grassroots doctors lack opportunities to timely understand and master the latest clinical guidelines and rational drug use norms from the outside world, as well as the awareness of continuous learning.

### 4.2 The problem of excessive medication in primary healthcare institutions is severe

According to the research results, the issue of unreasonable expenditure on medication costs caused by overuse is quite serious. The medication purchase list brought back by the standardized patients shows that most of these medication costs are spent on auxiliary and nutritional medications, primarily traditional Chinese medicine. When faced with common patient conditions such as postpartum depression, pediatric diarrhea, and stress urinary incontinence, doctors frequently prescribe adjuvant medications alone to patients. These traditional Chinese medicinal decoctions generally do not have strong or specific targeted efficacy. Even if ‘over-prescribed’ or ‘overused’, they will not cause significant harm to the human body, thus becoming a ‘disaster zone’ for excessive medication.

Although the use of traditional Chinese medicine in primary care has been shown to be ‘excessive’, in the process of exploring and discussing the reasons behind this, it has also triggered our researchers to think about this dialectically. Considering the regional culture, when faced with certain minor diseases, the grassroots areas sometimes have a preference for the use of traditional Chinese medicines when providing medical treatment programmes, which is a unique medication habit in China’s grassroots areas (37). Considering the health economy, traditional Chinese medicines tend to be more affordable, and they have a broad public base in grassroots clinics, which helps to promote the development of the local traditional Chinese medicine industry, and continues to contribute to the benefits of the health economy (38). Considering the attributes of medicines, Chinese medicines are mostly made from natural materials and have fewer side effects than Western medicines. While Western medicines focus on the precise targeting of a particular disease, proprietary Chinese medicines focus more on restoring the overall health of the human body and stimulating the body’s self-healing ability by improving the internal environment of the human body, which is why Chinese medicines have a better compatibility than Western medicines (37). The above discussion helps to help us dialectically view the role of Chinese medicines in contributing to over-medication, enabling us to take a more balanced view of the impact of Chinese medicines on over-use and healthcare expenditure.

### 4.3 Overuse is an essential factor leading to the increase in medical expenses

Empirical results indicate that excessive medical treatment, over-examination, and over-prescription all contribute to an increase in total medical expenses. While the addition of each unnecessary medical service may appear to cause only a small increase in medical costs, this increase still exceeds the reasonable growth level of medical expenses. Furthermore, marginal effect analysis reveals that these excessive medical services are not ‘high-priced individual items’ but rather consist of ‘multiple low-cost yet unnecessary’ services. In primary healthcare institutions, medical providers often adopt a ‘small but frequent’ approach to increase service volume and induce patient demand. If the total medical cost does not exceed the patient’s psychological expectation of consumption, such demand induction may likely be acquiesced by the patient or even unnoticed due to the asymmetric information between doctors and patients. The resulting medical expenses are ultimately borne by the patient. Since standardized patients were instructed to inform doctors that they would be paying out-of-pocket during visits, the generation of unnecessary medical expenses in this study is likely due to doctors prescribing additional medical services to obtain more revenue.

## 5 Suggestions and initiatives

### 5.1 Establish a sound standardized medical service system for primary healthcare institutions

In the current situation where overuse is more common in primary healthcare institutions, the Shaanxi Provincial Healthcare Commission has formulated a work plan for developing healthcare standardization. Standardization leads to the high-quality development of primary healthcare services, standardization promotes the balanced layout of medical resources in county hospitals, and standardization enhances the scientific and standardized management of primary hospitals, ultimately achieving the goal of reducing the incidence of overuse in primary healthcare institutions.

### 5.2 Strengthening the promotion of disease clinical guidelines and drug use norms

In the face of excessive examination and excessive use of medicines in excessive medical treatment, health administrative departments should regularly organize clinical guideline training for primary medical staff, encourage medical staff to continuously learn the latest clinical guideline knowledge, and improve clinical guideline compliance. At the same time, a special supervision team should be established to regularly inspect the diagnosis and treatment behaviors of primary medical institutions, and strengthen the supervision of pharmacological management and standardized use of drugs in primary medical institutions.

### 5.3 Optimizing the remuneration system for medical personnel in primary medical institutions

Gradually try to abolish the linking of medical personnel’s bonuses and wages to income from medicines, medical examinations and other businesses. Adhering to the service concept of ‘patient-centredness’, a performance appraisal system has been established with the quality of medical services, patient satisfaction, and reasonable diagnosis and treatment as the core indicators, and rewards have been given to medical personnel with outstanding performance, to motivate them to provide reasonable and high-quality medical services.

### 5.4 Strengthening residents’ health education on medical behaviors

With the help of online new media platforms and offline community bulletin boards, residents are educated on the identification and judgment of excessive medical treatment. Popular science articles and videos on reasonable medication and reasonable examination are released to popularize the knowledge of reasonable medical treatment, guide patients to set up the correct concept of medical treatment, and enhance residents’ awareness of reasonable medical treatment.

## 6 Research limitations

### 6.1 Lack of continuity in observation

This study only involved the initial interaction between standardized patients and the treating physicians. However, in actual clinical practice, patients and doctors may engage in continuous medical activities to achieve the ultimate goal of treating the disease. In continuous medical activities, the impact of over-medical treatment on costs may yield different results. For future research on over-medical treatment in continuous medical activities, due to the limitations of standardized patients, we will consider collecting information through retrospective analysis of real patients.

### 6.2 Lack of inpatient form investigation

Primary healthcare services consist of both outpatient and inpatient services, but this study solely focused on investigating the overtreatment in outpatient medical activities. To comprehend the full picture of overmedicalization in primary healthcare institutions, further research incorporating inpatient services is necessary, considering both outpatient and inpatient perspectives.

### 6.3 Limitations of research experience in promotion

In this study, Shaanxi Province, a region in western China, was selected as the target sample area for research and study. The overall socio-economic development level, primary healthcare service level and total population of Shaanxi Province rank in the middle of all Chinese provinces, so the findings of this province as the sample area of this study can reflect to some extent some of the common problems of Chinese provinces at the middle level of development. However, at the same time, we also need to take into account the differences between provinces. Shaanxi Province has a better overall socio-economic and healthcare development among the western regions of China, and its indicators, such as the number of beds, healthcare ratio, number of primary healthcare organizations, and per capita healthcare expenditure, are among the top of the other provinces in western China. At present, we have not yet conducted any research on other provinces in the western region, which have certain differences and disparities in the distribution of medical resources, ethnic minority concentration and human living habits. Therefore, in the future, based on the findings of the existing study, a research design that takes into account the geographic, economic, medical, and demographic distribution and characteristics of other provinces may be able to better extend the research experience and recommendations to other provinces in western China, provide a more comprehensive understanding of the current situation of primary overmedicine in the western region, and make more appropriate recommendations.

## Data Availability

The raw data supporting the conclusions of this article will be made available by the authors, without undue reservation.
